# Weighted Implication Grid: a graph-theoretical approach to modeling psychological change construction

**DOI:** 10.3389/fpsyg.2025.1630920

**Published:** 2025-10-03

**Authors:** Alejandro Sanfeliciano, Luis Angel Saúl, Luis Botella

**Affiliations:** ^1^Facultad de Psicología, Universidad Nacional de Educación a Distancia (UNED), Madrid, Spain; ^2^FPCEE Blanquerna, Universidad Ramon Llull, Barcelona, Spain

**Keywords:** graph theory, constructivist psychology, psychological change, psychometrics, Weighted Implication Grid (WimpGrid)

## Abstract

**Introduction:**

Anticipation and meaning-making are foundational processes in Personal Construct Psychology. Over the years, methodologies such as the Repertory Grid and Implication Grid have provided valuable tools for examining the anticipatory structure of personal meaning systems. Building on this tradition, the Weighted Implication Grid (WimpGrid) introduces a graph-theoretic and algebraic formalization of personal construct systems, aiming to enhance the modeling of psychological change as a dynamic and networked process.

**Method:**

The WimpGrid is based on a semi-structured interview in which participants evaluate hypothetical transformations in their self-perception across a set of personal constructs. These anticipatory judgments are recorded in a numerical matrix and formalized as a weighted directed graph, where nodes represent constructs and edges quantify the perceived influence between them. From this structure, graph-theoretical indices are derived to examine properties such as construct centrality, system dynamics, and resistance to change.

**Applications:**

WimpGrid enables idiographic assessment in clinical settings, supporting case formulation, therapeutic planning, and longitudinal monitoring of psychological transformation. Additionally, it provides a formalized methodological platform for research into subjective change processes and personal meaning structures.

**Discussion:**

By combining constructivist interviewing techniques with graph-theoretical modeling, WimpGrid offers a structured and flexible framework for investigating psychological change. It complements existing constructivist methodologies by providing tools for the quantitative analysis of complex meaning systems, and opens new avenues for theoretical refinement and empirical application.

## 1 Introduction

The evolution of the human mind has unfolded within a dynamic and uncertain environment, wherein the capacity to comprehend and anticipate events may have conferred an adaptive advantage for survival. This premise is central to Personal Construct Theory, which posits that “a person's processes are psychologically channelized by the ways in which he anticipates events” (Kelly, 1955, p. 32). This view is corroborated by Piaget (1954), who argued that individuals construct their understanding of the world through processes of assimilation and accommodation, thereby fostering adaptation. Similarly, Vygotsky (1978) emphasized that cognitive abilities—such as anticipation—emerge through social interaction, while Bruner (1990) highlighted the organizational role of narratives in the construction of meaning and the prediction of outcomes. Further advancing this line of reasoning, Maturana (1988) conceptualized knowledge as both relational and adaptive, demonstrating that our perception of reality is constituted through interaction, thereby facilitating both adaptation and anticipation in uncertain contexts. In this view, anticipation and meaning-making are core psychological operations that structure the personal theoretical framework by which individuals interpret the world and regulate their behavior and cognition.

Within the framework of Constructivism, anticipation is not conceptualized as a merely conscious projection of future events, but rather as the construction of meaning concerning them. Anticipation is thus seen as structurally isomorphic with the semantic system from which events are construed, irrespective of whether the events are explicitly imagined (Botella and Feixas, 1998). This epistemological stance aligns with the constructivist underpinnings of Kelly's theory, which assert that knowledge is actively built through subjective experience and interpretation. In this context, Hinkle (1965) sought to explore how individuals anticipate psychological change by investigating the inferential connections among personal meanings and identifying the resistances encountered in the process. This inquiry culminated in the development of his implication theory and the corresponding methodological tool: the Implication Grid (ImpGrid).

The present proposal builds upon Hinkle's foundational work, aiming to develop refined tools for analyzing the anticipatory mechanisms underpinning psychological change. Specifically, the Weighted Implication Grid (WimpGrid) integrates graph-theoretical modeling and a network perspective to quantify and mathematically formalize the structure of an individual's Personal Construct System (PCS). By doing so, it provides a robust analytical framework for representing the interrelations among constructs and their influence within a directed network. This approach is intended to enhance individuals' metacognitive insight into the strengths and limitations of their construct systems, thereby facilitating self-regulation and meaningful transformation aligned with personal goals.

### 1.1 Personal construct psychology

Personal Construct Psychology (PCP), originally formulated by Kelly (1955), offers a distinctive theoretical framework for understanding individuals through the system of personal constructs they use to interpret and anticipate their experiences. These constructs function as cognitive lenses, shaping the individual's engagement with their environment by organizing perception and guiding behavior. At the core of PCP lies the premise that individuals operate as intuitive scientists, continually revising their Personal Construct System (PCS) to enhance predictive accuracy and optimize adaptive functioning.

Kelly's theory is structured around a fundamental postulate and eleven corollaries (see Procter and Winter, 2020, p. 41–48) which together articulate a comprehensive epistemology of how individuals construct reality. The fundamental postulate asserts that “a person's processes are psychologically channelized by the ways in which he anticipates events,” highlighting the anticipatory and future-oriented nature of human cognition. Accordingly, perception, affect, and action are governed by the personal meanings assigned to anticipated experiences.

Among the eleven corollaries, the Organization Corollary is of particular importance for the current framework. It posits that individuals organize their constructs hierarchically, thereby structuring the PCS in a manner that facilitates efficient prediction and interpretation. This hierarchical structure allows individuals to classify and prioritize their experiences, supporting both the anticipation of future events and the regulation of adaptive responses. Such an organization is central to understanding how individuals conceive desired changes and experience resistance to transformation.

A principal psychometric challenge emerging from this epistemological stance is the development of methodological tools capable of measuring, modeling, and analyzing the PCS of individuals. Given the inherently idiographic orientation of PCP, which privileges individual meaning-making over the imposition of universal categories, effective psychological assessment must preserve the uniqueness and subjectivity of each construct system. This necessitates the design of methods that are both sensitive to individual variability and rigorous in their formalization and analysis.

The most extensively used method within PCP to access and study the PCS is the Repertory Grid Technique (RepGrid; Kelly, 1955). This semi-structured interview aims to elicit the structure of personal meaning by exploring how individuals construe themselves and significant others. It involves the construction of a rating matrix wherein a set of elements—typically roles of important individuals in the person's life—are evaluated across the respondent's personal constructs.

The RepGrid has made significant contributions to the understanding of psychological change within the framework of PCP. It has provided valuable methods for analyzing hierarchical organization, internal conflicts, and cognitive dilemmas that may inhibit personal transformation (see Bieri, 1955; Dorough et al., 2007; Feixas et al., 2000; Feixas and Saul, 2004; Landfield, 1977; Rouco et al., 2019; Sheehan, 1981). These analyses offer critical insights into the structural and dynamic properties of personal meaning systems and have proven especially useful in both clinical and research contexts.

Nevertheless, certain challenges arise when employing the RepGrid to directly assess the mechanisms underlying psychological change. According to the Range Corollary, personal construct systems are bounded in their applicability by the range of situations to which they are initially applied. In RepGrid protocols, the elicitation of constructs typically centers on significant others as elements, which–although central to interpersonal meaning-making–may not fully capture the evaluative structure relevant to the self or to aspirations for change. Additionally, RepGrid data is commonly analyzed using correlational techniques, which, while informative about structural consistency and inter-construct relationships, do not capture the directionality or conditional dependencies necessary to model change processes explicitly.

In light of these considerations, a range of complementary methodologies has emerged to deepen our understanding of the PCS and to explore its dynamic aspects with greater granularity (see Fransella, 2003). Among these, the ImpGrid stands out as a methodological advance that addresses some of the conceptual and analytical limitations associated with the RepGrid. Developed by Hinkle (1965), the ImpGrid draws on the corollaries of organization, modulation, and fragmentation, and shifts the analytic focus toward the inferential and hierarchical relations between constructs. This approach enables a more nuanced exploration of how constructs constrain or facilitate psychological change and allows for the identification of meaningful implications that structure personal meaning systems in non-linear and often non-reciprocal ways. Thus, the ImpGrid complements the RepGrid by extending the reach of constructivist assessment into the domain of anticipatory dynamics and systemic transformation.

### 1.2 Implication grid

What factors contribute to the resistance to change within personal construct systems? This foundational question led Hinkle (1965) to develop a theoretical and methodological framework for investigating the anticipatory dynamics that underlie psychological change. Building upon Kelly's fundamental postulate, Hinkle conceptualized resistance to change as contingent upon the individual's anticipations regarding that change. It is within this context that the notion of implication emerges–not merely as logical consequence, but as the anticipated reverberations within the PCS resulting from modifications to individual constructs. These implications define a network of semantic dependencies that organize and hierarchize the PCS, thereby structuring the individual's experiential reality.

To systematically study such anticipatory patterns, Hinkle developed the ImpGrid, a semi-structured interview technique wherein hypothetical changes in constructs are explored to elicit a matrix of anticipatory relationships. The resulting matrix reveals which constructs are perceived as influencing or being influenced by others in the context of potential change.

The ImpGrid methodology offers several notable advantages for the study of psychological transformation: (a) It centers on the individual's self and ideal self, thereby generating contextually rich and personally meaningful data related to change; (b) It enables the identification of both congruent and incongruent regions within the PCS, highlighting areas where change is expected versus those where resistance is likely; (c) It yields directional information, as the implication matrix explicitly represents the anticipated influence between constructs.

These characteristics represent an important evolution in the assessment of psychological change when compared to the Repertory Grid (RepGrid), which typically focuses on interpersonal roles rather than internal change dynamics, and infers construct relationships indirectly through correlational analysis. In contrast, the ImpGrid captures these interrelations directly and within an anticipatory framework, allowing for greater precision and interpretive clarity.

Despite these contributions, it is important to acknowledge certain methodological limitations that have motivated further developments in this domain. Interested readers are encouraged to consult the broader literature for a detailed evaluation of the ImpGrid's strengths and limitations (Fransella et al., 2004). Within the scope of the present work, we focus on two key limitations relevant to the formal analysis of psychological change.

First, implications in the ImpGrid are treated as binary (i.e., present or absent). While this dichotomous representation offers interpretive simplicity, it may fail to capture the gradational and probabilistic nature of actual psychological processes. As noted by Botella (2020), some implications are subjectively experienced with greater intensity or salience than others. Indeed, Hinkle himself acknowledged the scalar nature of psychological influence in his original thesis, suggesting that a more refined model could allow for graded, rather than categorical, relationships among constructs.

Second, the analytical procedures commonly used with ImpGrid data–primarily frequency counts and pattern distributions—lack the formal rigor required to extract structural insights from the full matrix of implications. As Bell (2014), Fransella et al. (2004), and Korenini (2016) have pointed out, these methods may obscure subtle but meaningful interdependencies within the PCS, and do not adequately reflect the system's underlying complexity. This highlights the need for a more sophisticated mathematical formalization capable of modeling the PCS as a dynamic and structured network of relationships.

The primary objective of this paper is to introduce the WimpGrid, a methodological innovation that addresses these two limitations while preserving the theoretical integrity and idiographic richness of the original ImpGrid. Like its predecessor, the WimpGrid is grounded in the principles of PCP and employs a semi-structured interview format. However, it incorporates key refinements: it assigns a continuous weight to each implication, thereby capturing the intensity of inferred relationships, and it leverages graph-theoretical and algebraic techniques to formalize the structure of the PCS. These advancements enable the construction of mathematical models that more accurately reflect the system's complexity and offer new avenues for the quantitative study of psychological change. The following sections provide a detailed exposition of the theoretical and methodological foundations of the WimpGrid.

### 1.3 Weighted Implication Grid

The name *Weighted Implication Grid* encapsulates the conceptual foundations of this methodology: (a) Grid, as it draws upon the interview-based structure and epistemological premises of PCP and the RepGrid (Kelly, 1955); (b) Implication, as it centers on the anticipatory relations between constructs, following Hinkle's formulation of implication as a key dynamic in psychological change (Hinkle, 1965); and (c) Weighted, to denote the model's capacity to represent the gradational and fuzzy nature of these implications, enabling the construction of mathematically precise representations of the PCS.

In light of these foundations, the WimpGrid represents a synthesis and extension of the conceptual framework proposed by Hinkle (1965), particularly with regard to his typology of psychological change. WimpGrid builds upon this legacy by introducing a weighted and mathematically formalized framework capable of capturing the underlying dynamics of personal meaning systems. Unlike models that assume strictly hierarchical construct arrangements, WimpGrid accommodates both hierarchical and non-hierarchical relationships. In doing so, it addresses Hinkle's own recognition that personal constructs often exhibit mutual implication and interconnectedness rather than unidirectional influence.

A key area of critique in Hinkle's original thesis relates to the assumption that constructs identified through laddering are inherently superordinate–a position that has been challenged by subsequent empirical and theoretical work showing that construct relations are frequently symmetrical or context-dependent (Bell, 2014; Caputi et al., 1990). Furthermore, the dichotomous nature of implication coding in the original ImpGrid has been noted to oversimplify the dimensional character of psychological experience (Korenini, 2016). The WimpGrid addresses these concerns by adopting a weighted, graph-based perspective in which constructs are modeled as nodes in a directed network with weighted edges, reflecting the intensity and directionality of anticipatory influence. This formalism allows for the modeling of psychological change as a continuous and graded process, rather than as a binary or discrete phenomenon.

The principal contribution of the WimpGrid lies in its ability to approach the subjectivity of psychological change through a rigorous mathematical lens. By integrating structured qualitative interviews with algebraic and graph-theoretical formalization, the WimpGrid provides a versatile framework for analyzing the PCS. This methodology offers several key advantages:

1. A precise assessment of the diffuse and graded implications within the PCS, capturing both the categorical nature of construct polarity and the gradual dynamics of psychological change. This is essential for modeling the anticipated trajectory of psychological transformation in a way that aligns with the lived experience of change.
2. A standardized mathematical model that effectively encodes subjective perspectives, facilitating clear communication between patient and clinician. This shared model also supports clinical anamnesis by preserving construct records over time, offering a comprehensive map of the patient's evolving PCS.
3. A network-based view of constructs as part of a dynamic and interconnected system. The WimpGrid's graph-theoretical tools reveal underlying PCS properties, such as conflict cycles and tension points, providing insights into the complex structural patterns within an individual's construct system.
4. An empirical framework that opens new avenues for research into psychological change. By leveraging a network approach, the WimpGrid refines our understanding of personal constructs, enhancing both theoretical insights and clinical applications.

In summary, the WimpGrid advances Hinkle's foundational insights by addressing key methodological limitations and introducing a formal apparatus capable of representing the complexity of personal meaning systems. Its capacity to model weighted relationships among constructs marks a significant development in constructivist psychometrics, with implications for both clinical and research contexts. Recent empirical work illustrates its applicability: Botella et al. (2022) and Saúl et al. (2023) have employed WimpGrid in clinical case formulation and supervision, while Saúl et al. (2022) used it to assess motivational structure in habit change. Ongoing research initiatives documented in the Open Science Framework (Sanfeliciano and Saúl, 2025a; Saaúl et al., 2024) further demonstrate its versatility in tracking psychological change across time and contexts.

These developments underscore the WimpGrid's potential as both a clinical instrument and a scientific methodology–one that offers a mathematically grounded, yet deeply personalized, window into the structure of psychological experience.

## 2 WimpGrid implementation

**Figure 1:**
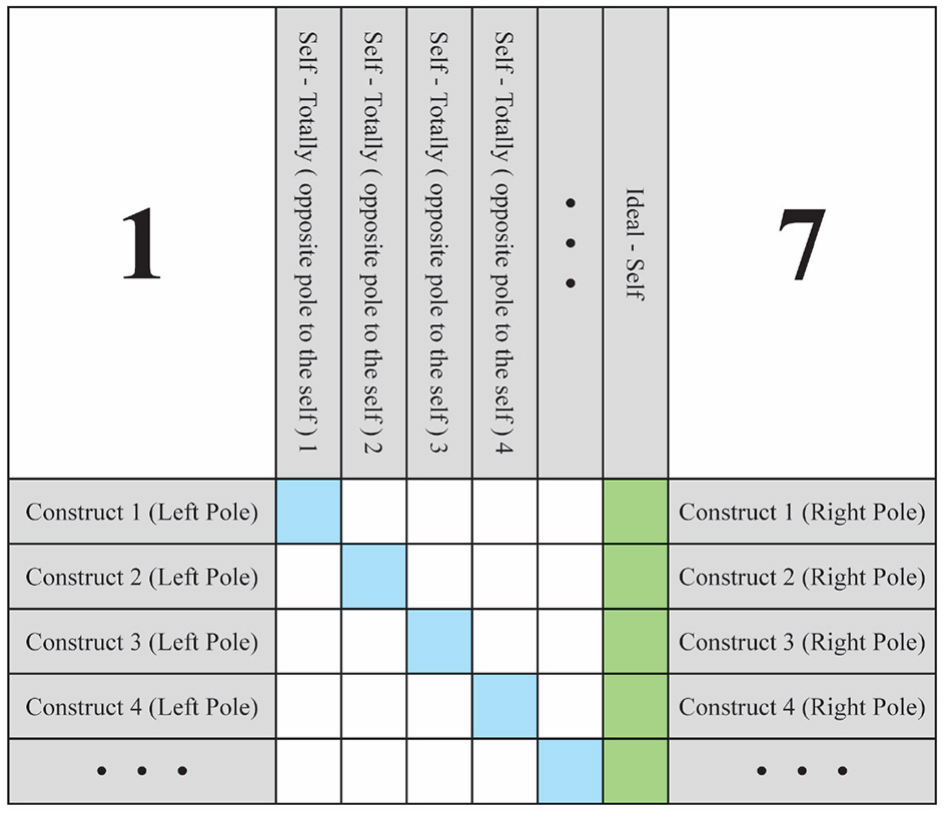
Weighted Implication Grid template.

Understanding the WimpGrid protocol requires a clear outline of its structure and procedural logic. The WimpGrid is a semi-structured interview designed to explore how changes in one area of a person's self-concept may influence other psychological dimensions. To do this, the respondent is asked to imagine specific, significant changes in their personality or functioning, and then reflect on how these changes would affect other parts of their identity. The responses are recorded in a structured template (Figure 1), which facilitates the systematic analysis of the participant's personal meaning system.

The template contains several key components. First, the personal constructs—dimensions the participant uses to describe themselves (e.g., “calm vs. anxious”)—are placed in the rows. The columns represent various imagined versions of the self, referred to as *Hypothetical-Selves*, each corresponding to a major change in one construct. The *Self-Now* ratings, highlighted in blue, are located along the diagonal and reflect how the participant currently sees themselves. The final column, highlighted in green, corresponds to the *Ideal-Self*, or how they would ideally like to be. The remaining cells are used to capture the participant's judgments about how a hypothetical change in one construct would influence their experience of the others.

### 2.1 Initial setting

The first step is to identify a set of personal constructs that are meaningful to the participant. These constructs can be elicited through a variety of methods such as triadic comparisons, self-characterizations, or open interviews (see Fransella et al., 2004; Hinkle, 1965; Keen and Bell, 1980; Kelly, 1955; Neimeyer et al., 2002). The chosen constructs should be relevant to the participant's desired psychological changes and reflect both personal goals and perceived difficulties.

Once the constructs are selected, the participant evaluates themselves on each construct in two ways: Self-Now and Ideal-Self. These ratings are made using a scale that best suits the context–commonly a 1 to 7 scale, where 1 represents one pole of the construct and 7 the opposite. Although other typical scales include [1,3] and [1,9]. For a more detailed examination of the distinctions between the various scales, I would suggest consulting the work of Metzler et al. (2002).

Next, for each construct, a Hypothetical-Self is defined. This is an imagined version of the self where a major change has occurred in that specific construct. The idea is to take the participant as far as possible from their current self in that dimension.

- If the *Self-Now* is defined at one end of the spectrum, the *Hypothetical-Self* will be the most extreme score at the other end. To illustrate, if we consider the *Calm-Anxious* construct and the individual considers themselves to exhibit mild anxiety, their Hypothetical Self for that construct will be characterized as *Self-Totally Calm*. The following rules guide this definition:
- In the event that the *Self-Now* is not defined at either pole (intermediate score), but the *Ideal-Self* is, the most extreme score oriented in the same direction as the Ideal will be used. To illustrate, if we consider the construct *No sporty-Sporty* and the subject is situated between the two poles, yet their ideal is to be sporty, their hypothetical self will be *Self-Totally Sporty*.
- If both the *Self-Now* and *Ideal-Self* ratings are located at the midpoint of the scale (e.g., 4 on a 1–7 scale), indicating no clear preference toward either pole, the construct will be arbitrarily assigned to the right pole to define the hypothetical self.

**Figure 2:**
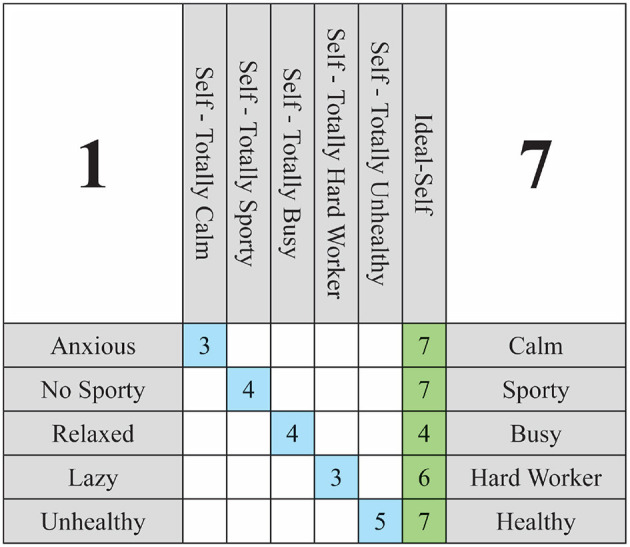
Weighted Implication Grid 5 × 5 example.

In accordance with this criterion, the objective is to posit a hypothetical self for each construct that is as distant as possible from the individual's current self in that construct. This helps generate a stark contrast when presenting hypothetical situations, resulting in scenarios that are both cognitively significant and more readily imaginable. Figure 2 provides an illustrative example of a 5 × 5 grid with all hypothetical constructs defined. Readers are encouraged to examine the relationship between the *Self-Now*, the *Ideal-Self*, and each of the *Hypothetical-Self* in the figure. It is important to note that the main diagonal of the WimpGrid matrix is reserved for representing the Self-Now ratings. This decision reflects a mathematical convention that facilitates the calculation of differential changes across constructs. The hypothetical versions of the self are labeled verbally in the column headers (e.g., “Self–Totally Calm”), but their values are not numerically displayed in the grid. Instead, they are internally modeled as projections to the extreme poles of the standardized scale (i.e., to +1 or -1 in the vector $\vec{h}$). This distinction between visual representation and computational modeling helps maintain clarity for the participant's self-assessment, while enabling consistent transformation and inference of directional influence throughout the system.

### 2.2 Grid scoring

Once the preliminary preparations have been concluded, we may then proceed to administer the WimpGrid interview and complete the scoring matrix. The protocol to be followed is:

1. The first construct is selected from the respondent's list.
2. We pose the hypothetical situation for the selected construct. We explain how to do this later in the text.
3. The interviewee is told their *Self-Now* score and asked about changes they anticipate as a result of the situation.
4. Note any changes in the *Hypothetical-Self* column. If there are no changes, note the *Self-Now* score. If there are changes, note the new score given by the respondent.
5. Select the next construct and repeat these steps until there are no constructs left.

In establishing a hypothetical situation, it is essential to ensure that the message is unambiguous and accurately reflects the objective of the evaluation. The following is an exemplar of a speech:

Let's evaluate the causal implications of your meaning (selected construct). Earlier you indicated to me that you felt defined by (pole associated with self). Think for a moment about what (selected construct) means to you and what it would be like if you were to switch to the other pole, that is, if you woke up one morning and realized that you described yourself best when you defined yourself as totally (pole opposite to self). What would your new self be like after you switched from (pole associated with self) to totally (pole opposite self in the selected construct)? Remember that the change in (selected construct) is the cause, while the changes in the rest of the meanings are the effects, and these effects only result from the change from being (pole associated with self) to being totally (pole opposite self).

**Figure 3:**
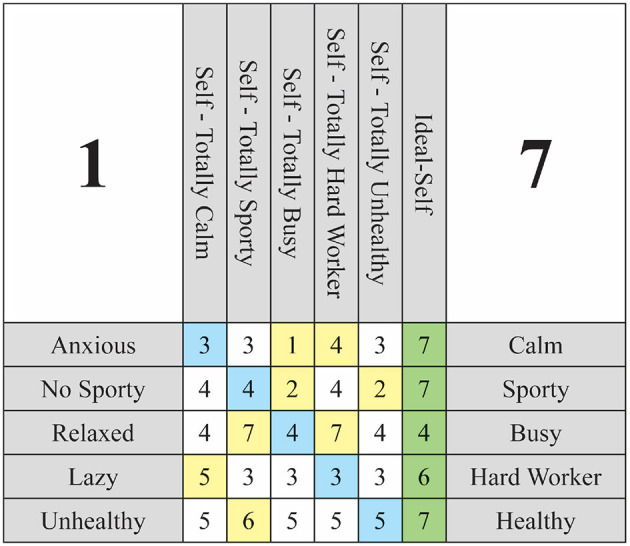
Weigthed Implication Grid 5 × 5 example with all scores.

Following this explanation, the template must be presented to the individual undergoing assessment and the column corresponding to the hypothetical situation must be completed. It is crucial for the interviewee to be aware of their initial scores on the *Self-Now* and to modify them accordingly to align with the hypothetical situation. At this juncture, it may be beneficial to utilize a visual aid, such as cards or a computer visualization, to assist the interviewee in recognizing all of their constructs and the associated scores for their *Self-Now*. Upon conclusion of the interview, we should obtain a completed grid as illustrated in Figure 3, wherein the alterations asserted by the interviewee are highlighted in yellow.

### 2.3 Key considerations in the application of the WimpGrid

The effective application of the WimpGrid requires attention to several methodological considerations that can influence the quality and interpretability of the data. Among these, the number and nature of personal constructs included in the interview play a critical role. While larger grids may offer greater analytical depth, they can also introduce respondent fatigue and reduce data quality. It is therefore recommended to prioritize the elicitation of a manageable number of psychologically salient constructs—those that are personally meaningful and relevant to the domain of change under investigation. Constructs should not only reflect the respondent's lived experience but also show potential interconnectivity; disconnected or tangential constructs may obscure the structure of the PCS and reduce analytical coherence.

Another crucial element is the correct identification of implication directionality. During the interview, participants are asked to consider hypothetical changes to specific constructs and their anticipated effects on the self. It is essential that they interpret these scenarios causally (i.e., “If this construct were to change, how would that affect you?”), rather than inversely (“What would I need to change to achieve that construct?”). Misinterpretation of this direction can introduce systematic error into the weight matrix. To safeguard against this, interviewers should prompt participants to verbally justify their responses, thereby allowing the direction of implication to be explicitly verified in real time.

Equally important is the differentiation between direct and indirect implications. The WimpGrid methodology assumes that implications recorded during the interview refer to immediate, direct influences between constructs. However, respondents may inadvertently refer to indirect or mediated pathways. For instance, if a respondent claims that being more relaxed would lead to better health, but their reasoning involves becoming more sporty as an intermediate step, then the direct link from relaxation to health is inferentially invalid. Overlooking such chains may lead to the overestimation of certain relationships. To mitigate this, interviewers should actively explore the logic behind each response, clarifying whether the connection is direct or mediated, and adjust the implication network accordingly.

Finally, the semi-structured nature of the WimpGrid must be preserved to ensure that the data reflect the participant's unique cognitive architecture. The interview should not be treated as a rigid procedural script; rather, it should function as a flexible conversational framework that promotes reflection and adaptation. Given the metacognitive demands of the task, interviewers must be prepared to support participants in articulating and refining their responses. This adaptive stance enhances the ecological validity of the data and ensures that the resulting network of implications faithfully represents the respondent's subjective meaning system.

In summary, the methodological rigor of the WimpGrid is not solely grounded in its mathematical formalism but equally in the quality of the interaction through which data is elicited. Careful construct selection, clarification of implication directionality, validation of directness, and flexible, reflective interviewing are all essential components for producing meaningful and analytically robust representations of the PCS.

## 3 Mathematical foundations

The WimpGrid provides a mathematically formalized framework to model the relationships and dynamics within an individual's PCS. This approach leverages graph theory and algebra to quantify psychological constructs, supporting a deeper understanding of self-perception, desired change, and system relationships.

Let [−1, 1]^*n*^ denote the *n*-dimensional normalized psychological space, where *n* is the number of personal constructs elicited during the WimpGrid interview, and each coordinate represents one such construct. Each score within this space is bounded in the interval [−1, 1], ensuring a standardized representation of subjective meanings. A value of −1 indicates complete identification with the left pole of the construct, whereas a value of 1 denotes full alignment with the right pole. Intermediate values correspond to partial associations, reflecting varying degrees of semantic positioning between the two poles. This modeling framework is supported by four key mathematical components that formalize the individual's current self-perception, ideal self, the set of hypothetical situations elicited during the interview, and the anticipated systemic effects across constructs.

- *Self-Now Vector (*$\vec{s}$*)*: The individual's current self-perception is represented as a vector $\vec{s}\in {\left[-1,1\right]}^{n}$, where each component *s*_*i*_ corresponds to their score on construct *i*, for *i* = 1, …, *n*. This normalized continuous scale allows for nuanced differentiation in self-assessment across all constructs.
- *Ideal-Self Vector (*$\vec{d}$*)*: The ideal self is encoded as a vector $\vec{d}\in {\left[-1,1\right]}^{n}$, where *d*_*i*_ denotes the desired value on construct *i*. This vector serves as a referential anchor to evaluate psychological discrepancy and the directionality of intended change.
- *Hypothetical Situations Vector (*$\vec{h}$*)*: This vector $\vec{h}\in {\left\{-1,1\right\}}^{n}$ represents the direction of each hypothetical self-transformation, with components *h*_*i*_ defined as:

(1) $$h_{i}=\{\begin{array}{ll} -\text{sgn}(s_{i}), & \text{if }s_{i}\neq 0,\\ \text{sgn}(d_{i}), & \text{if }s_{i}=0\text{ and }d_{i}\neq 0,\\ 1, & \text{if }s_{i}=0\text{ and }d_{i}=0. \end{array}$$

This vector encodes the direction in which each construct would shift under a maximally contrasting hypothetical situation, taking values at the extremes of the normalized scale.

- *Hypothetical-Selves Matrix (M)*: The matrix *M*∈[−1, 1]^*n*×*n*^ encodes the perceived state of all constructs under hypothetical changes. Each off-diagonal entry *m*_*ij*_ reflects how a transformation in construct *i* is expected to influence construct *j*, while the diagonal elements *m*_*ii*_ store the baseline self-perception values from $\vec{s}$.

All four components described above are derived from raw participant ratings (e.g., [1, 7], [1, 9]). To ensure comparability across scales and support the mathematical formalism, each raw score *r* is linearly transformed into a normalized value *x*∈[−1, 1] using the following equation:


(2) $$\begin{array}{l} x=2\cdot \frac{r-r_{\text{min}}}{r_{\text{max}}-r_{\text{min}}}-1 \end{array}$$

This transformation ensures that all constructs are placed within a common reference frame, preserving their directional structure while enabling the algebraic operations that follow. Here, *r*_min_ and *r*_max_ denote the minimum and maximum values of the original rating scale.

A fundamental aspect of the WimpGrid mathematical model is its assumption of proportionality in the relationships between constructs. Specifically, changes in one construct (Δ*s*_*i*_) are linearly related to the changes they induce in another (Δ*s*_*j*_):


(3) $$\begin{array}{l} \Delta s_{j}=w_{ij}\Delta s_{i} \end{array}$$

Using the data gathered during the interview, Δ*s*_*j*_ and Δ*s*_*i*_ can be calculated as follows:


(4a) $$\begin{array}{l} \Delta s_{j}=m_{ji}-s_{j} \end{array}$$

(4b) $$\begin{array}{l} \Delta s_{i}=h_{i}-s_{i} \end{array}$$

Based on this proportionality assumption, the weight matrix *W*, a core component of WimpGrid analysis, is derived as:


(5) $$\begin{array}{l} w_{ij}=\frac{m_{ji}-s_{j}}{h_{i}-s_{i}} \end{array}$$

Each element *w*_*ij*_ is a scalar that captures the sensitivity of construct *j* to a hypothetical change in construct *i*. Intuitively, a weight *w*_*ij*_ is calculated as the magnitude of change in construct *j* divided by the magnitude of change in construct *i* that caused it. It expresses the proportion of change observed in construct *j* relative to the hypothetical change in construct *i*, and quantifies how strongly a change in one construct is expected to influence another.

It is important to note that the estimation of *w*_*ij*_ may be affected by ceiling or floor effects when the Self-Now score *s*_*j*_ is already close to the scale's minimum or maximum. In such cases, the observable change (*m*_*ji*_−*s*_*j*_) may be limited, potentially underestimating the influence of construct *i* on construct *j*. This is a known limitation of models operating over bounded rating scales (Arslan and Benke, 2023; Liu and Wang, 2021; Twisk, 2023). However, it may also reflect a meaningful psychological interpretation: individuals who perceive themselves as already close to one extreme may implicitly consider further change in that direction as less relevant or less probable. To address this, we are currently exploring the use of nonlinear transformation functions or open-ended rating formats to enhance the sensitivity and expressiveness of the model in such edge cases.

This *W* matrix serves as the basis for constructing a structural map of the PCS through the application of Graph Theory. Constructs are represented as nodes, with directed edges illustrating relationships of influence. Each edge is weighted by *w*_*ij*_, while nodes carry attributes such as the Self-Now (*s*_*i*_) and Ideal-Self (*d*_*i*_) scores. The model transforms the weight matrix *W* into a directed graph *G* = (*V, E*, ψ, φ), where:

- *V*: Represents the constructs as nodes.
- *E*: Denotes directed edges, which correspond to non-zero weights (*w*_*ij*_) in the matrix *W*.
- ψ: Maps attributes such as the Self-Now (*s*_*i*_) and Ideal-Self (*d*_*i*_) scores to each node.
- φ: Assigns edge weights, capturing the influence of one construct on another.

The WimpGrid's mathematical formulation transforms subjective constructs into analyzable patterns, bridging individual narratives and empirical rigor. By representing personal meanings as a network of interconnected constructs, it opens the door to exploring critical psychological phenomena such as centrality, resistance to change, and alignment between self-perception and goals.

### 3.1 Analysis

The calculation of the *W* matrix enables the performance of diverse mathematical analyses, which facilitate a deeper comprehension of the PCS of the evaluee. Such analyses include the examination of digraphs, centrality of meanings, conflicting implications, and the dynamics that hinder psychological change. These results can be obtained with the R package WimpTools (Sanfeliciano and Saúl, 2025b) and from the web application PsychLab (https://psychlab.uned.es), which has been developed by the UNED Constructivist Research Group (GICUNED).

#### 3.1.1 Digraphs

**Figure 4:**
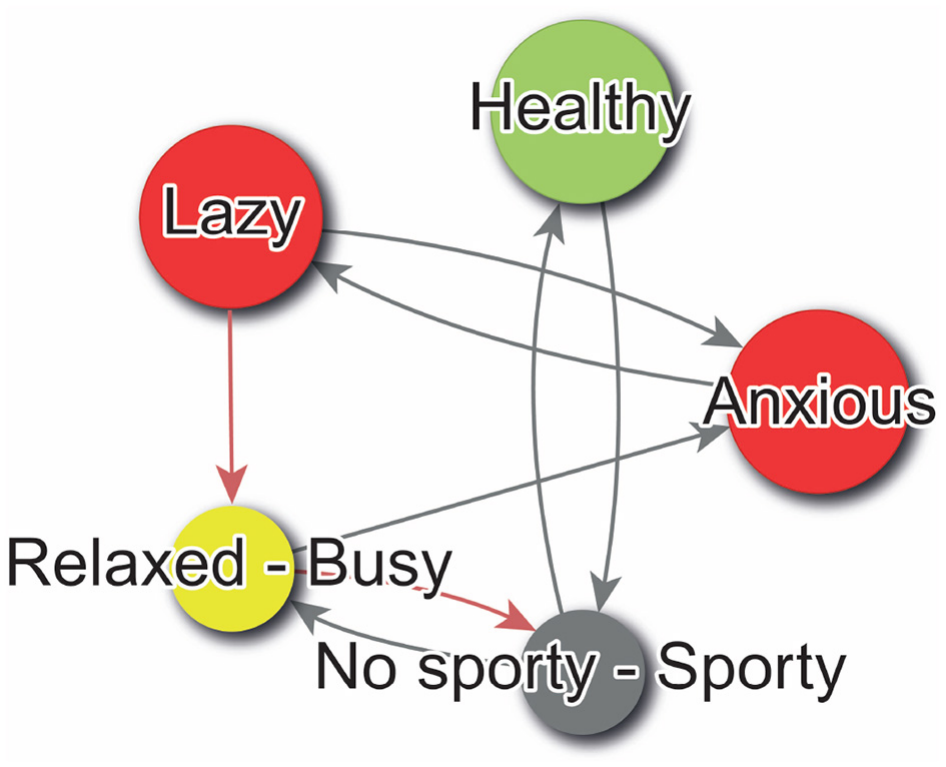
Sample digraph create with WimpTools (Sanfeliciano and Saúl, 2025b).

The directed graph, otherwise known as a digraph, is the optimal representation for elucidating the implications of PCS. This is a specific type of graph, in which the edges are associated with a directionality. This implies that the interconnections between the graph's vertices are oriented in a particular direction, thereby enabling the representation of unidirectional relationships between constructs. To generate this representation, we may utilize our *W* matrix as if it were a weighted adjacency matrix, following the methodology delineated in Wasserman and Faust (2013, p. 147-161). Figure 4 illustrates an example of a digraph that can be constructed using the *W* matrix.

In the digraphs generated with WimpTools (Sanfeliciano and Saúl, 2025b) and PsychLab, a color code supports the interpretation of the psychological system. Node colors indicate the type of alignment between *Self-Now* and *Ideal-Self* : green nodes represent *congruent constructs*—those where both self and ideal share the same polarity, indicating alignment in meaning; red nodes indicate *discrepant constructs*—where the self and ideal are located on opposite poles, reflecting psychological conflict; yellow nodes correspond to *dilemmatic constructs*—where the *Ideal-Self* score is in the midpoint of the scale, suggesting indecision or ambivalence about what is desirable. Gray nodes indicate constructs where the *Self-Now* score lies in the midpoint of the scale, and thus lacks a clear definition; in such cases, both poles are displayed in the node label to reflect this ambiguity, and the right-hand pole is used for determining the direction of hypothetical changes and their implications for the rest of the construct system. Edge colors represent the type of perceived implication between constructs: black edges denote direct relationships, where movement toward the pole of construct *i* increases the presence of construct *j* (i.e., both change in the same direction), while red edges indicate inverse relationships, where movement toward construct *i* implies a decrease in construct *j* (i.e., opposite direction). Only the poles that define the self (as indicated in the *Self-Now* vector) are shown in the node labels, except in undefined cases as previously described. Finally, node size is proportional to the degree of self-definition, operationalized as the absolute value of the *Self-Now* rating |*s*_*i*_|; constructs with strong identity relevance (i.e., ratings near the scale extremes) appear larger in the visualization.

Referring to Figure 4, the subject self-identifies as healthy, aligning with their ideal in this regard. However, they also perceive themselves as lazy and anxious, which contrasts with their ideal self-image. Notably, there is no clear ideal associated with the Free-Busy construct, and the individual remains undecided between the poles of Sporty and No Sporty. Examining the digraph's edges reveals key relationships among constructs. For example, a positive feedback loop strengthens the link between Healthy and Sporty. Additionally, there is a direct association between being Busy and experiencing anxiety, with anxiety also contributing to laziness. A self-regulatory, or negative feedback loop, further connects Busy, Anxious, and Lazy constructs, among others. Together, these insights elucidate the structural dynamics of the individual's PCS.

With an understanding of graph theory and experience in graph analysis, it is possible to extract a great deal of interesting information from graphs. However, as the size of the graphs increases, it becomes increasingly challenging to analyse them visually. This is why a series of algorithms have been developed to generate different arrangements, which can be of significant assistance in the interpretation of digraphs. The mathematical field of graph theory is replete with a plethora of algorithms.

**Figure 5:**
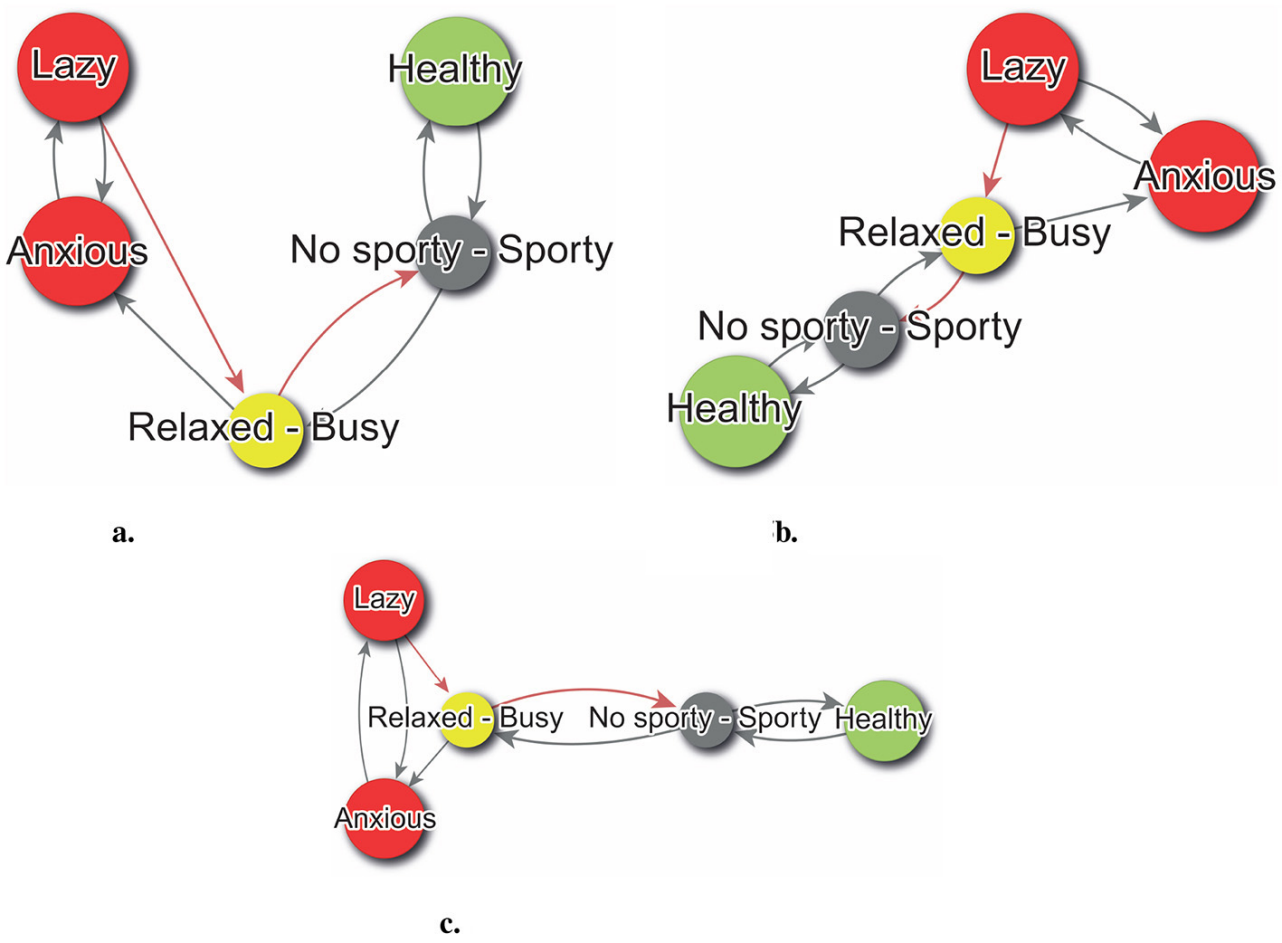
Digraphs layouts. **(a)** Reingold-Tilford. **(b)** Graphopt. **(c)** Multidimensional scaling.

This article presents three illustrative examples: first, proposed by Reingold and Tilford (1981), establishes a hierarchy to identify constructs with the most and least influence on the system, placing the most influential at the top and the least influential at the bottom (Figure 5a). The Graphopt algorithm, developed by Schmuhl (2003), arranges nodes to avoid overlap and groups highly connected constructs, aiding in understanding which constructs are most impacted by changes, as illustrated in Figure 5b. Lastly, multidimensional analysis, based on Quetelet's work (Peña, 2013), arranges elements on a plane according to similarity, providing insights into the similarity between constructs by their proximity in the layout, as shown in Figure 5c.

#### 3.1.2 Centrality measures

An additional analytical procedure that can be carried out with the aid of the weight matrix is the calculation of centrality measures. These indices indicate the degree of involvement of each node in the existing network dynamics. This information is crucial for determining whether the constructs within the system are more or less prominent. As clinicians or researchers, these measures facilitate the identification of pivotal and central constructs, thereby enhancing our comprehension of the underlying dynamics of psychological change.

The degree centrality measure is the most intuitive for graph-based networks, representing the number of edges connected to each vertex. In digraphs, such as those resulting from the application of the weight matrix, it is possible to quantify the number of inputs and outputs associated with each construct. The highest number of outputs will indicate the greatest influence within the system, while the highest number of inputs will indicate the greatest level of influence exerted.

In order to calculate the degree, given an weight matrix *W* of dimension *n*×*n*, where *n* is the number of constructs, the set of Equations 6 is used to calculate the total incoming and outgoing influence of each construct, expressed as the weighted in-degree ($k_{i}^{-}$) and out-degree ($k_{i}^{+}$), respectively. These are computed as the sum of all incoming and outgoing weights for node *i*:


(6a) $$\begin{array}{l} k_{i}^{-}=\overset{n}{\underset{j=1}{\sum }}w_{ji} \end{array}$$

(6b) $$\begin{array}{l} k_{i}^{+}=\overset{n}{\underset{j=1}{\sum }}w_{ij} \end{array}$$

(6c) $$\begin{array}{l} k_{i}=k_{i}^{-}+k_{i}^{+} \end{array}$$

According to the hierarchical framework originally proposed by Hinkle (1965), constructs can be classified as *superordinate* or *subordinate* based on the directionality of their implicative relationships—those that tend to generate more implications are considered superordinate, while those that are more often affected are subordinate. However, studies by Bell (2014) and Korenini (2016) have highlighted limitations in this strictly hierarchical view. Bell argues that Hinkle's structure, based on unidirectional implications, may oversimplify the complexity and reciprocity often present in construct systems. Similarly, Korenini emphasizes that a purely hierarchical model may obscure constructs that are interdependent or function at multiple systemic levels.

To address these limitations, the Presence-Balance index (PB; Sanfeliciano et al., 2025) was proposed as a more flexible representation of construct connectivity and influence. This index is derived through a linear transformation (Equation 7) of the input and output degrees of constructs, producing two components: *Presence* (*P*_*i*_) and *Implication Balance* (*B*_*i*_). The transformation employs the orthonormal factor $0.5\sqrt{2}$ to rotate the space by 45°, ensuring that both components are expressed on a comparable scale and can be interpreted independently.


(7) $$\begin{array}{l} PB_{i}=\left(P_{i},B_{i}\right)=\left(\begin{array}{cc} 0.5\sqrt{2} & 0.5\sqrt{2}\\ -0.5\sqrt{2} & 0.5\sqrt{2} \end{array}\right)\left(\begin{array}{c} k_{i}^{-}\\ k_{i}^{+} \end{array}\right) \end{array}$$

This model retains the spirit of Hinkle's superordinacy concept while extending it by incorporating both the total implication connectivity and the directional balance of each construct. The *Presence* index quantifies the overall level of implication (inputs and outputs), identifying broadly connected constructs. Meanwhile, the *Implication Balance* captures the hierarchical tendency–whether a construct acts predominantly as an originator (superordinate) or as a recipient (subordinate) of implications.

**Figure 6:**
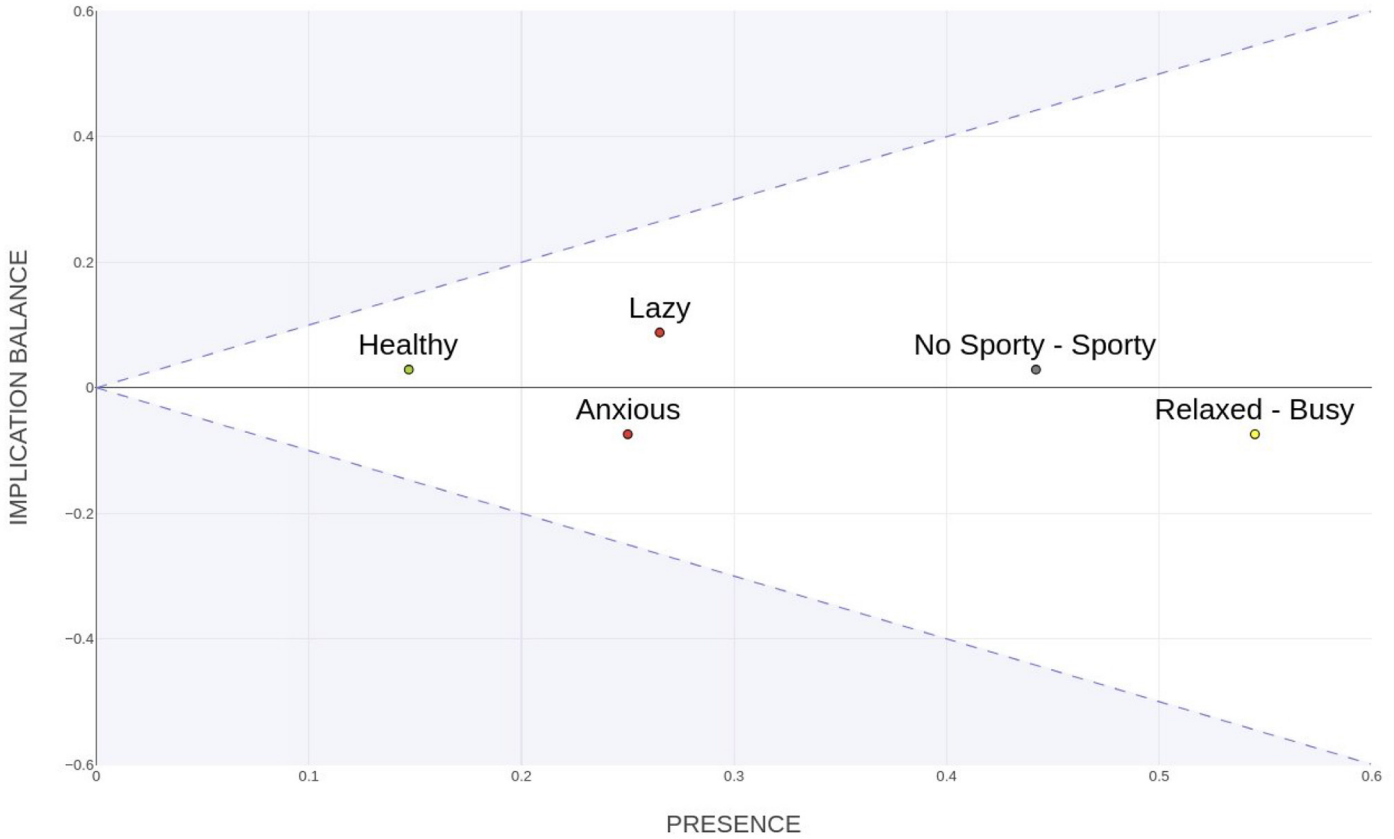
Plot of PB index.

The vectorial nature of the PB index allows constructs to be represented in a two-dimensional space, with the horizontal axis reflecting overall connectivity and the vertical axis indicating systemic role. This approach addresses the limitations noted by Bell (2014) and Korenini (2016), allowing for a more nuanced representation of systemic influence. Figure 6 illustrates this space, where shaded regions denote mathematically unfeasible zones, emphasizing the interdependence between presence and hierarchy. Thus, the PB graph extends Hinkle's theoretical foundation, integrating network centrality into a constructivist framework of psychological structure.

#### 3.1.3 Change to ideal implications

One analytically rich avenue within the framework of the WimpGrid is the examination of weighted implications that emerge when a construct incongruent with the Ideal-Self exerts influence over other constructs. This type of configuration may reveal underlying psychological conflicts, particularly when such influence constrains or threatens other constructs that are already aligned with the ideal. This conceptualization is related to the notion of implicative dilemmas in PCP, which describe situations where a desired change in one construct is anticipated to produce an undesired shift in another (see Dorough et al., 2007; Feixas et al., 2000; Feixas and Saul, 2004).

Unlike traditional approaches that rely on correlational patterns between self-discrepant and congruent constructs to detect such dilemmas, the WimpGrid leverages the directionality and magnitude encoded in the weight matrix *W*. This allows for a finer-grained analysis of anticipatory dynamics, as it provides information not only about the co-occurrence of constructs but about how change in one is expected to affect another.

**Figure 7:**
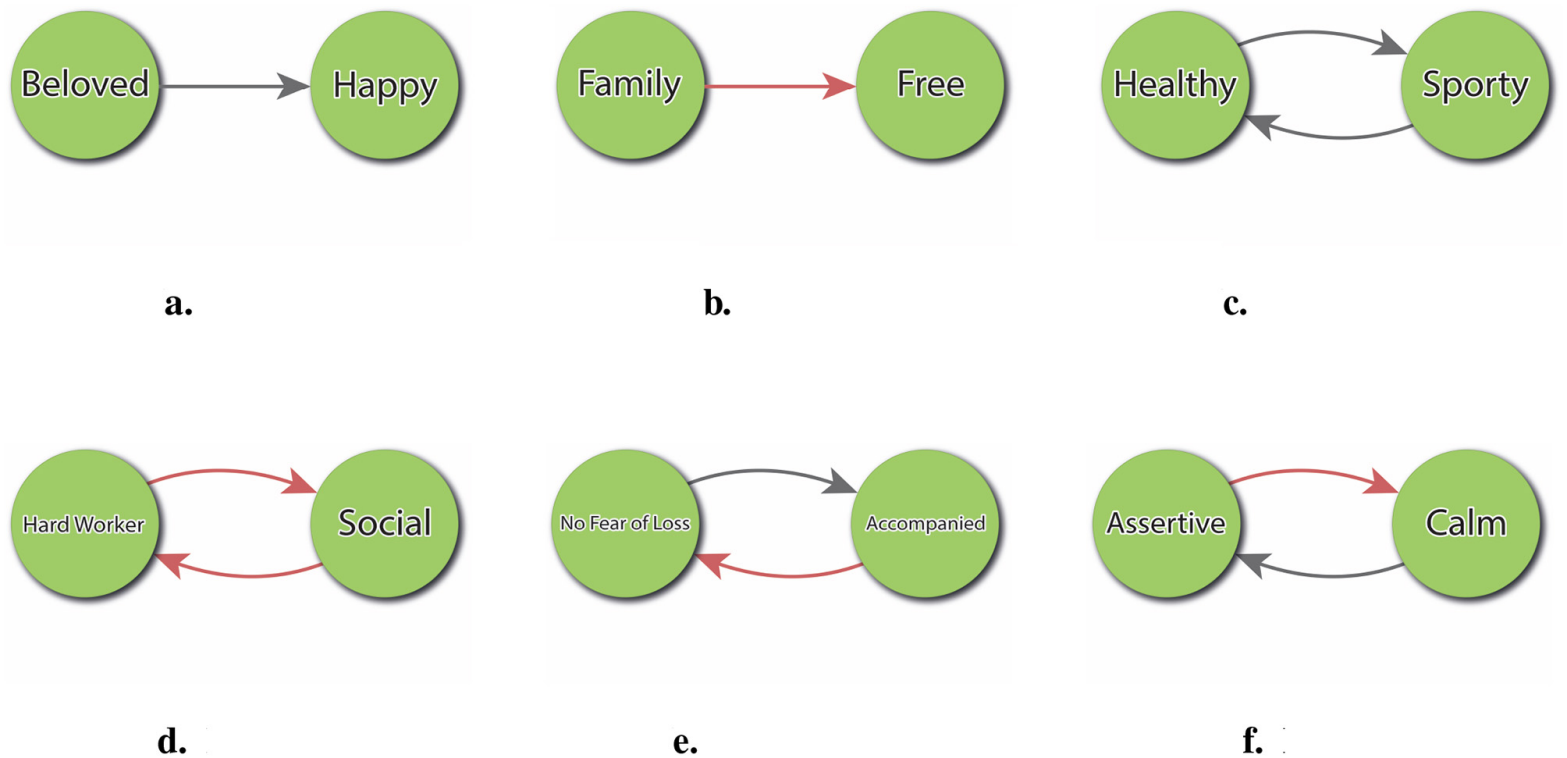
Typology of change to ideal implications. **(a)** Positive impact without feedback. **(b)** Negative impact without feedback. **(c)** Positive impact with positive feedback. **(d)** Negative impact with positive feedback. **(e)** Positive impact with negative feedback. **(f)** Negative impact with negative feedback.

These implications can be formally analyzed through pairwise evaluations in the directed graph representation of the PCS. Each pair of constructs may exhibit one of several possible relational patterns, depending on their respective alignment with the Ideal-Self and the direction of their mutual influence. As illustrated in Figure 7, six such implication types can be distinguished. This classification facilitates the identification of potential synergies–such as mutually reinforcing constructs like Sporty and Healthy–as well as anticipated conflicts, such as when striving to become more Hardworker is expected to reduce Social Life.

Such analysis not only enhances our understanding of the structural organization of meaning systems, but also provides valuable clinical insight into ambivalences and motivational tensions that may affect psychological change.

The analysis of pairwise relationships provides interesting information; however, the study of the set of all implications that act on a discrepant construct allows for the exploration of deeper concepts. These include: (a) *Impact*, which represents the consequences that a person anticipates in their system when they imagine a change toward the ideal in a given construct; and (b) *Feedback*, which shows the subject's anticipation of the feedback that this change receives from the system. This approach allows us to gain a comprehensive understanding of the anticipated consequences and feedback associated with a change from a global perspective. To operationalise this index, denoted IF, the set of Equation 8 may be employed. In this calculation, a weight matrix oriented with respect to the ideal (W^*^) is necessary, in which the poles of each construct are aligned to satisfy the directionality imposed by the Ideal-Self vector.


(8a) $$\begin{array}{l} IF_{i}=\left(I_{i},F_{i}\right) \end{array}$$

(8b) $$\begin{array}{l} I_{i}=\overset{n}{\underset{j=1}{\sum }}w_{ij}^{*} \end{array}$$

(8c) $$\begin{array}{l} F_{i}=\overset{n}{\underset{j=1}{\sum }}w_{ij}^{*}w_{ji}^{*} \end{array}$$

The IF index provides clinically meaningful insight into the internal coherence and anticipated consequences of striving toward a desired self. Unlike simple discrepancy measures, which merely identify aspirational gaps, the IF index reveals whether a person's psychological system supports or resists the realization of a specific ideal. For instance, in Figure 7c illustrates a construct (*Healthy*) whose ideal pole is mutually reinforced with another positively valued construct (*Sporty*), forming a cycle of congruent expectations. This kind of configuration is typically experienced as motivationally coherent and emotionally empowering. In contrast, Panel 7d depicts a more problematic configuration: the individual anticipates that becoming a *Hard Worker* (ideal change) will lead to being less *Social* (negative impact), but also expects that being less *Social* will in turn reinforce being a *Hard Worker* (positive feedback). This circularity may result in a self-reinforcing cycle where the person moves further away from valued social engagement, making the change both psychologically costly and systemically entrenched. Such dynamics are invisible in simple analyses, but are made explicit through the IF index. This allows clinicians to detect patterns of hidden resistance or systemic traps that may undermine well-intentioned change goals.

While the IF index offers a powerful summary of the anticipated impact and feedback of ideal-directed change, it is still grounded in the analysis of direct relationships. However, meaning systems often exhibit emergent dynamics that unfold through indirect and recursive pathways. The psychological effects of change can ripple through constructs over time, producing secondary or delayed responses that are not immediately evident. To capture these higher-order dynamics, it is necessary to model the propagation of activation across the network structure of the personal construct system. The next section develops a formal dynamic model based on activation flow and feedback cycles, enabling simulation of how hypothetical change in one construct reverberates through the entire system.

#### 3.1.4 System dynamics

An understanding of the dynamics of a system is critical to fully grasp the interrelationships between its constituent constructs. While systems with few vertices and edges can be interpreted at a glance, complex networks often obscure patterns and dependencies that require mathematical modeling for proper analysis. To this end, dynamic simulations serve as an indispensable tool.

A common approach to analyzing such systems involves activation propagation: assigning numerical values to nodes and allowing these activations to spread through the weighted edges. This procedure, inspired by Fuzzy Cognitive Maps (Felix et al., 2019; Kosko, 1986; Tsadiras, 2008; Wang and Mendel, 1992), provides insights into how changes in one construct ripple through the network over time.

Our implementation in WimpTools follows this tradition and defines a discrete-time dynamical system with decay and threshold-limited activation. Each simulation involves three components: (a) an initial activation vector, (b) a propagation rule based on the weight matrix, and (c) a thresholding function that constrains the activation values to the original rating scale. The equations governing the system dynamics are shown below:


(9a) $$\begin{array}{l} \forall t\in \mathbb{N},\;\vec{s}\left(t+1\right)=f_{th}\left(\vec{s}\left(t\right)+\vec{a}\left(t\right)\right) \end{array}$$

(9b) $$\begin{array}{l} \forall t\in {\mathbb{N}}^{+},\;\vec{a}\left(t\right)=W^{T}\Delta \vec{s}\left(t\right)e^{-\lambda t} \end{array}$$

(9c) $$\begin{array}{l} \forall t\in {\mathbb{N}}^{+},\;\Delta \vec{s}\left(t\right)=\vec{s}\left(t\right)-\vec{s}\left(t-1\right) \end{array}$$

(9d) $$f_{th}(x)=\{\begin{array}{ll} -1 & \text{if }x\leq -1\\ x & \text{if }-1<x<1\\ 1 & \text{if }x\geq 1 \end{array}$$

Here, $\vec{s}\left(t\right)$ is the state of the system at time *t*, $\vec{a}\left(t\right)$ is the activation input at time *t*, and *W*^*T*^ is the transpose of the weight matrix. The term *e*^−λ*t*^ introduces a decay factor, with λ∈[0, 1] controlling how quickly the influence of previous changes diminishes (A typical choice is λ = 0.1). The threshold function *f*_*th*_(·) ensures that activations remain within the bounded scale [−1, 1] (Equation 9d).

The simulation is initialized by defining the vector of external activation $\vec{a}\left(0\right)$, which represents an initial perturbation or intervention applied to the system. This activation is applied to the current self-perception vector $\vec{s}\left(0\right)$, typically corresponding to the Self-Now ratings. In most cases, $\vec{a}\left(0\right)$ is configured to affect a single construct (e.g., “lazy - hard worker”) by assigning it a positive or negative activation value, while the remaining dimensions are set to zero. This setup models a targeted intervention or a spontaneous shift in self-perception and allows the subsequent system dynamics to be computed iteratively as the influence propagates through the network.

**Figure 8:**
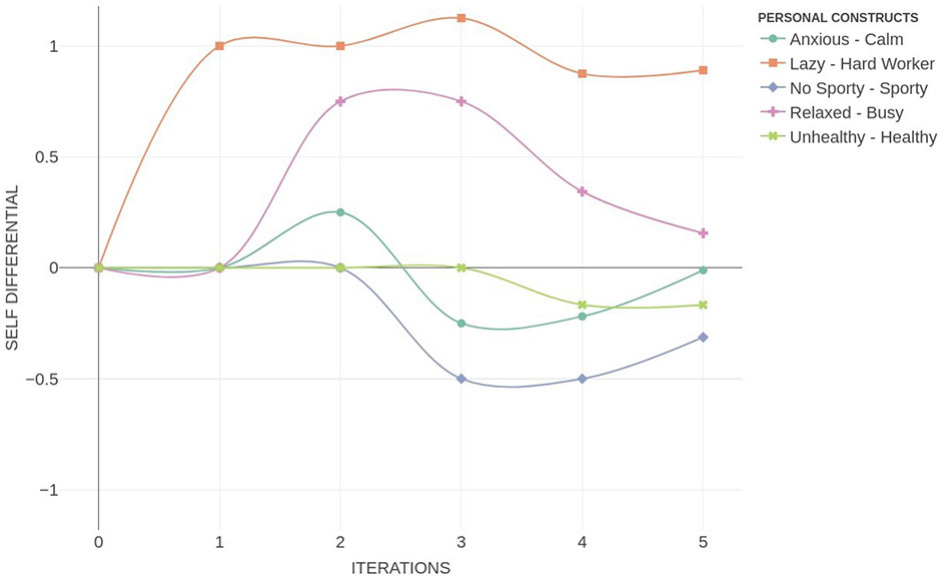
Personal construct system dynamics plot.

Figure 8 illustrates the temporal evolution of a PCS. The x-axis represents discrete time steps *t*, while the y-axis denotes the differential activation levels of each construct *i* relative to their initial state, specifically *s*_*i*_(*t*)−*s*_*i*_(0). Each trajectory captures the dynamic propagation of a hypothetical change through the construct network, revealing patterns of amplification, attenuation, or temporal delay. This representation enables a nuanced analysis of how anticipatory shifts reverberate across the PCS structure.

This form of simulation enables practitioners to visualize how an individual anticipates the systemic consequences of adopting a particular personal construct. In the case illustrated in Figure 8, the hypothetical scenario of becoming a “Hard Worker” initiates a dynamic trajectory of change throughout the personal construct system. The simulation shows that this shift leads to an anticipated increase in “Busy”, suggesting that the person links hard work with heightened activity or workload. Subsequently, this change triggers an activation of “Anxious”, indicating a perceived emotional cost associated with this anticipated change. Interestingly, this ripple effect extends further, exerting a subtle negative influence across additional constructs, which gradually deviate from their initial self-position. This illustrates how a seemingly desirable change (e.g., working harder) may be internally associated with stress or imbalance. Such patterns—otherwise difficult to infer from static representations—can inform practitioners about the anticipatory logic underpinning the client's meaning system, and help identify which constructs act as amplifiers of conflict or targets for psychological intervention.

#### 3.1.5 Reproducibility and software availability

All analyses and visualizations presented in this manuscript can be fully replicated using the open-source WimpTools package for R. The package includes the dataset example.wimp, which corresponds exactly to the illustrative case used throughout the manuscript. This dataset, together with the package's core functions, provides access to all key components of the analysis. Users can reproduce all figures, run dynamic simulations, and compute the proposed indices directly within the WimpTools environment, ensuring transparency, replicability, and ease of methodological adoption.

The package is freely available for download from both the Zenodo repository (Sanfeliciano and Saúl, 2025b) and the official GitHub repository (https://github.com/GICUNED/WimpTools).

## 4 Discussion

The way individuals construct their experiential world fundamentally determines how they engage with processes of psychological change. When change is contemplated, the first obstacle often arises not from external circumstances, but from the internal anticipatory mechanisms through which the individual interprets that change. Consequently, understanding resistance or openness to transformation requires methodological tools capable of capturing and analyzing the anticipatory architecture of the personal meaning system. The WimpGrid addresses this psychometric challenge by offering a methodology that allows for an analysis of psychological change from an ideographic perspective, grounded in Hinkle (1965) theories on personal constructs.

As demonstrated, the WimpGrid enables a formal and detailed mathematical analysis of key structural features within the PCS, such as construct centrality, systemic dynamics, and internal conflicts. This tool advances the study of psychological change by framing it not as a sequence of discrete adjustments, but as a reconfiguration within a complex, interconnected system of personal meanings. Through the application of graph theory—long established as a robust framework for modeling complex relational systems—the WimpGrid reconceptualizes implications between constructs as weighted, directional edges within a cognitive network. This network-based perspective opens new avenues for understanding both the structural facilitators and the implicit constraints individuals face as they move toward change.

One of the persistent challenges in constructivist psychology lies in articulating subjective meaning systems in a form that is analyzable, communicable, and clinically useful. Personal constructs are inherently idiographic, which complicates standardization without compromising psychological depth. The WimpGrid contributes to this challenge by providing a formalized and scalable language for representing the subjective architecture of meaning. By doing so, it offers a promising step toward enabling more consistent interpretation of individual constructs across therapeutic and research contexts, while preserving the complexity and uniqueness of each person's experiential world. However, we recognize that these methodological innovations should be understood as proposals–formally grounded, but still under empirical development–rather than definitive solutions to long-standing challenges in the field. The WimpGrid provides a structured framework for engaging with these challenges, and its effectiveness will depend on the accumulation of further evidence and refinement through practice.

The methodological properties of the WimpGrid render it particularly well-suited for both clinical and empirical applications. In psychotherapy, it supports case formulation by revealing the anticipatory structures that shape client difficulties (Botella et al., 2022), and provides a consistent framework for documenting therapeutic insights. In clinical supervision (Saúl et al., 2023), it allows for dynamic tracking of therapeutic progress by visualizing the evolution of the PCS over time. During intervention, the WimpGrid promotes cognitive defusion and reflective awareness by engaging individuals in an exploration of their own self-justifications and recurring conflict patterns (Saúl et al., 2022). In research contexts, it functions as both a psychometric instrument and a formal model that integrates individual variability into a mathematically analyzable structure. This dual capacity makes it a valuable tool for advancing our understanding of psychological change and the internal constraints individuals may face in pursuing personally meaningful transformations.

### 4.1 Limitations and future directions

While the WimpGrid offers substantial methodological and theoretical advantages, several limitations must be considered prior to its broader application. These include: (a) the cognitive demands associated with the interview procedure, (b) the reactivity inherent in the data collection process, and (c) the epistemological and technical assumptions embedded in its mathematical modeling of subjective meaning. Each of these issues warrants careful examination.

The first limitation concerns the cognitive complexity of the WimpGrid interview, which requires participants to engage in sustained metacognitive activity by responding to hypothetical scenarios. As Iarossi (2006) notes, such tasks may exceed the introspective capacity of some individuals, particularly when responses involve events not directly experienced. This can result in inconsistencies or speculative answers, potentially compromising data validity.

Despite these demands, the use of hypothetical selves remains theoretically central to the constructivist tradition. As Kelly (1955) emphasized, “a person's processes are psychologically channelized by the ways in which he anticipates events.” Even imagined anticipations reveal emotionally salient goals and internalized standards that shape motivation and openness to transformation. Accordingly, the interviewer plays a crucial role in scaffolding the reasoning process and minimizing bias. Software tools such as PsychLab and WimpTools may further enhance this process by providing structured interfaces and adherence to protocol.

In order to assess the empirical robustness of the WimpGrid, a recent validation study has examined its psychometric properties using a Multitrait-Multimethod design across three core psychological domains: Emotional Adjustment, Centrality of Meaning, and Resistance to Change (Sanfeliciano and Saúl, 2025a). Conducted with a within-subjects design (*N* = 87), the study compared WimpGrid responses against those from Repertory Grids and standardized self-report instruments. The results revealed strong test-retest reliability ($r_{xx^{\prime }}=0.92$) and significant convergent validity for key indices. These findings suggest that WimpGrid is capable of capturing stable and theoretically meaningful aspects of the anticipatory structure of the self. Its mathematical modeling framework, grounded in graph theory, supports the extraction of clinically relevant indices that reflect dynamic and systemic psychological properties. Although these data are part of an ongoing line of research and remain unpublished, full access to the preregistration, methods, and results is available via the OSF repository. This initial evidence provides a promising empirical foundation for the WimpGrid as a psychometric tool for modeling psychological change.

A second limitation relates to the phenomenon of reactivity: the possibility that participation in the WimpGrid interview may itself influence the structure of the PCS being assessed. As participants are prompted to articulate and evaluate implications among their constructs, they may undergo shifts in their personal meaning system during the very process of its measurement. While this challenges conventional notions of psychometric stability, it also aligns with the core premise of Personal Construct Psychology–that meaning-making is dynamic and self-revising (Winter, 2013). From a clinical standpoint, such reactivity may be viewed not as a limitation but as an opportunity: the WimpGrid can serve as a tool for therapeutic reflection, fostering insight, self-awareness, and psychological reorganization (Procter and Winter, 2020).

The most fundamental limitation, however, lies in the level of abstraction inherent in any mathematical representation of psychological processes. As with all formal systems, the WimpGrid is constructed upon simplifying assumptions–namely, that personal meanings can be modeled as scalar, linear, and directionally weighted entities within a graph-theoretic framework. While these assumptions may not capture the full richness and contextual variability of subjective experience, they constitute a necessary and appropriate starting point. The development of scientific models typically begins with parsimonious formulations that enable analytical tractability and theoretical clarity. From this foundation, future research can progressively refine the model by incorporating more complex features–such as non-linear dynamics, probabilistic dependencies, or temporal evolution–based on empirical evidence. In this sense, the WimpGrid offers not only a functional and interpretable structure for studying personal meanings, but also a flexible platform for ongoing theoretical and methodological advancement.

In summary, the Weighted Implication Grid offers a structured approach to the assessment and modeling of psychological change within a constructivist framework. By combining idiographic depth with formal mathematical representation, it contributes to bridging the gap between subjective meaning systems and analytical modeling. While it is not without limitations, the WimpGrid provides a coherent platform for exploring the anticipatory structure of personal constructs and their role in change processes. Its capacity to formalize internal dynamics such as resistance, conflict, and motivational direction opens up new avenues for both clinical practice and psychological research. As empirical work continues and methodological refinements are introduced, the WimpGrid may serve as a useful tool for advancing our understanding of how individuals construe themselves and their possibilities for transformation.

## Data Availability

The original contributions presented in the study are included in the article/supplementary material, further inquiries can be directed to the corresponding author.
